# Tyrosine–Chlorambucil Conjugates Facilitate Cellular Uptake through L-Type Amino Acid Transporter 1 (LAT1) in Human Breast Cancer Cell Line MCF-7

**DOI:** 10.3390/ijms21062132

**Published:** 2020-03-20

**Authors:** Piman Pocasap, Natthida Weerapreeyakul, Juri Timonen, Juulia Järvinen, Jukka Leppänen, Jussi Kärkkäinen, Jarkko Rautio

**Affiliations:** 1 Division of Pharmaceutical Chemistry, Faculty of Pharmaceutical Sciences, Khon Kaen University, Khon Kaen 40002, Thailand; piman.pocasap@kkumail.com; 2 Human High Performance and Health Promotion Research Institute, Khon Kaen University, Khon Kaen 40002, Thailand; 3 School of Pharmacy, Faculty of Health Sciences, University of Eastern Finland, P.O. Box 1627, FI-70211 Kuopio, Finland; juri.timonen@uef.fi (J.T.); juulia.jarvinen@uef.fi (J.J.); jukka.leppanen@uef.fi (J.L.); jussi.karkkainen@uef.fi (J.K.)

**Keywords:** AT1, cancer, chlorambucil, cellular uptake, antiproliferative, MCF-7

## Abstract

l-type amino acid transporter 1 (LAT1) is an amino acid transporter that is overexpressed in several types of cancer and, thus, it can be a potential target for chemotherapy. The objectives of this study were to (a) synthesize LAT1-targeted chlorambucil derivatives and (b) evaluate their LAT1-mediated cellular uptake as well as antiproliferative activity in vitro in the human breast cancer MCF-7 cell line. Chlorambucil was conjugated to l-tyrosine—an endogenous LAT1 substrate—via either ester or amide linkage (compounds **1** and **2**, respectively). While chlorambucil itself did not bind to LAT1, its derivatives **1** and **2** bound to LAT1 with a similar affinity as with l-tyrosine and their respective cellular uptake was significantly higher than that of chlorambucil in MCF-7. The results of our cellular uptake study are indicative of antiproliferative activity, as a higher intracellular uptake of chlorambucil derivatives resulted in greater cytotoxicity than chlorambucil by itself. LAT1 thus contributes to intracellular uptake of chlorambucil derivatives and, therefore, increases antiproliferative activity. The understanding gained from our research can be used in the development of LAT1-targeted anticancer drugs and prodrugs for site-selective and enhanced chemotherapeutic activity.

## 1. Introduction

l-type amino acid transporter 1 (LAT1), commonly referred to as the large neutral amino acid transporter 1, is a Na^+^-independent amino acid transporter responsible for the transport of mainly large and neutral amino acids from extracellular fluids into cells. LAT1 substrates include several essential amino acids, e.g., leucine, isoleucine, valine, tryptophan, methionine, histidine, tyrosine, and phenylalanine [1]. In addition, LAT1 is also known to mediate the transport of thyroid hormones and prescription drugs (e.g., melphalan, gabapentin, and l-dopa). LAT1 has also been demonstrated to transport amino acid-containing prodrugs, in which amino acids as promoieties have been linked with non-substrate parent drugs. The prodrug strategy has been exploited, for example, to utilize LAT1 as a drug carrier to increase drug permeability through the blood–brain barrier by conjugating various drugs (e.g., dopamine, ketoprofen, and valproic acid) to various amino acids [2,3,4].

High levels of LAT1 are expressed in many cancerous tissues [5], the overexpression being a result of the higher amounts of essential amino acids needed to support the growth and development of the malignancy [6]. LAT1 is thus an intriguing target for improved and targeted tumor therapy [7]. Some LAT1 inhibitors have, on the other hand, been developed to thwart the supply of amino acids, impeding protein synthesis and cancer cell proliferation [8,9,10]. Recently, a LAT1 inhibitor JPH203 displayed promising results in a clinical phase I study, especially in patients with advanced solid tumors expressing high levels of LAT1 [11,12]. The results emphasized LAT1 as a potential target for cancer treatment. On the other hand, only a very few anticancer agents have been modified to take advantage of LAT1-targeted cancer cell uptake [13].

**Figure 1:**
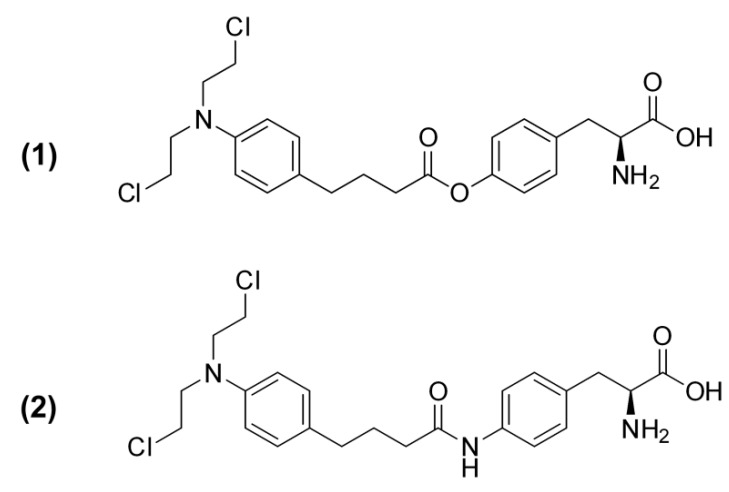
Structures of chlorambucil derivatives **1** and **2.**

The chemotherapeutic agent chlorambucil is mainly used in the treatment of chronic lymphocytic leukemia as well as some types of lymphoma; however, the cancer resistance mechanisms, i.e., efflux by MRP1 as well as drug metabolism by glutathione (GSH) conjugation and β-oxidation, reduce chlorambucil intracellular levels and hinder its effectiveness [14,15]. The objective of the present study was to modify the structure of chlorambucil so that it resembles LAT1 substrates, thereby enhancing its intracellular uptake through LAT1-mediated transport, and thus increasing its efficacy against cancer cells. Based on the LAT1 model [16], chlorambucil derivatives **1** and **2** (Figure 1) were designed: chlorambucil was linked from its carbonyl group with an endogenous LAT1 substrate, l-tyrosine, or its amine derivative by either an ester or amide bond, respectively. The affinity of chlorambucil and its derivatives to LAT1, their cellular uptake, and antiproliferative activity were determined in a LAT1-expressing human breast adenocarcinoma cell line (MCF-7). The obtained results support rational drug/prodrug design to increase drug uptake into cancer cells via a specific transporter and, thereby, enhance chemotherapeutic efficiency.

## 2. Results

### 2.1. Synthesis of Chlorambucil Derivatives

**Scheme 1:**
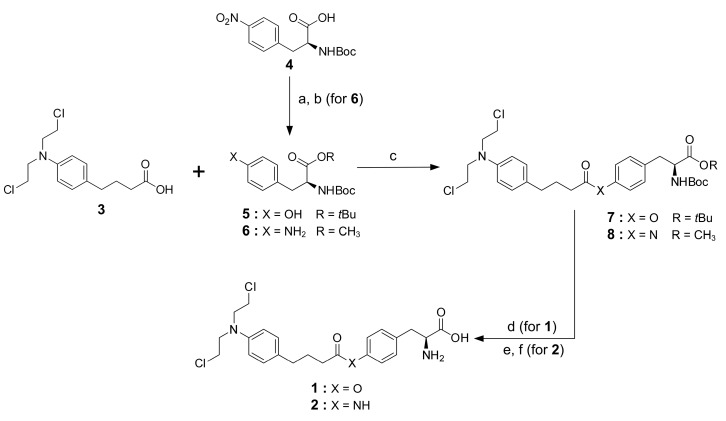
Synthetic pathway for chlorambucil derivatives. Reagents and conditions: (**a**) K_2_CO_3_, (CH_3_O)_2_SO_2_, acetone, overnight, and (**b**) 10% Pd-C, H_2_, MeOH, overnight; (**c**) EDC, DMAP, CH_2_Cl_2_, overnight; (**d**) TFA, CH_2_Cl_2_, 6 h; (For **2**) (**e**) LiOH, THF, 1.5 h, and (**f**) TFA, CH_2_Cl_2_, 2 h; all conditions were conducted at room temperature.

Compounds **1** and **2** were designed to link chlorambucil with tyrosine or its amine derivative through either an ester or an amide bond, respectively. Compound **1** was synthesized by conjugating chlorambucil, **3,** to commercially available Boc-l-tyrosine-O*t*Bu, **5,** using EDC/DMAP and then removing of the protecting groups (Boc and O*t*Bu) by trifluoroacetic acid. In compound **2** synthesis, the amino acid part (Boc-4-amino-l-phenylalanine methyl ester, **6**) was first synthesized as previously described [16] with methyl esterification of Boc-4-nitro-l-phenylalanine, **4,** using dimethyl sulfate in the presence of K_2_CO_3_. The reduction of the nitro group was done by hydrogenation in the presence of 10% Pd-C. Then, peptidic coupling of chlorambucil with amino derivative **6** was catalyzed by EDC/DMAP. Finally, deprotection of the protecting groups, i.e., methyl ester and Boc, using LiOH and trifluoroacetic acid, respectively, afforded chlorambucil derivative **2** (Scheme 1).

### 2.2. In Vitro Conversion

**Figure 2:**
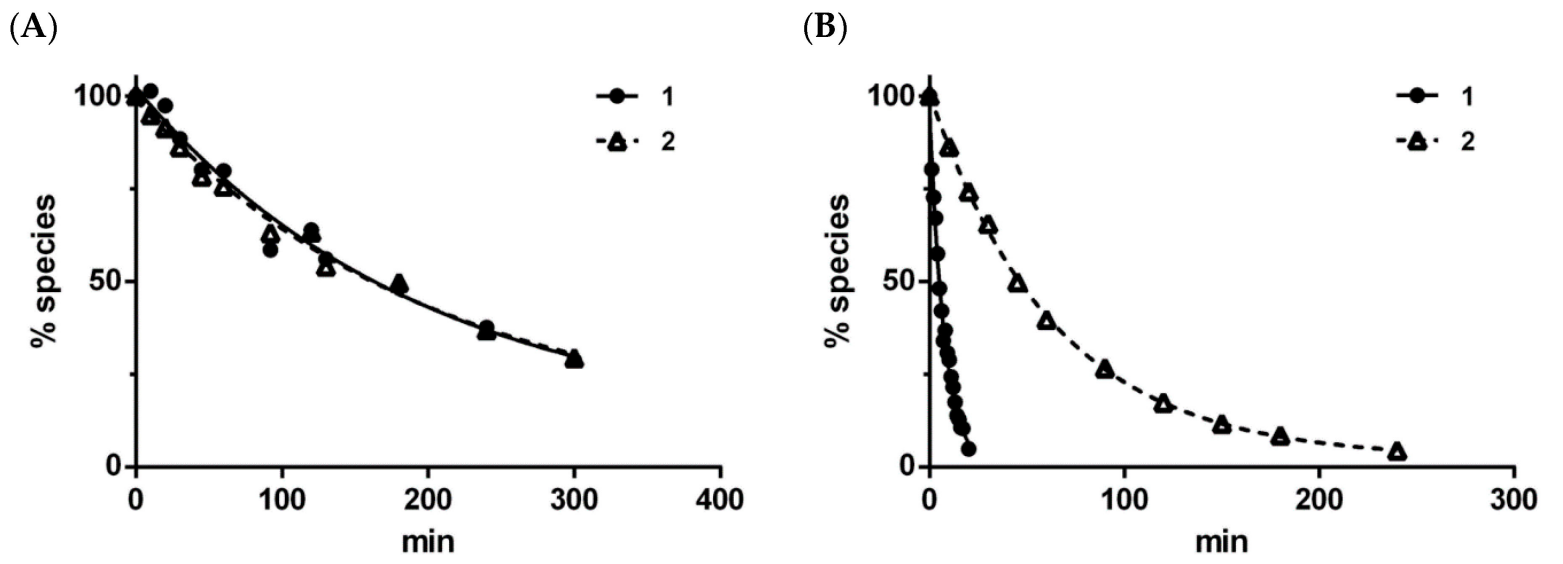
Time courses (mean, *n* = 2) for chlorambucil derivatives **1** and **2** during chemical conversion in phosphate buffer (pH 7.4) (**A**) and enzymatic conversion in human microsomes (pH 7.4) (**B**) at 37 °C.

The in vitro conversion of compounds **1** and **2** was determined both in aqueous buffer solution (pH 7.4) and in human liver microsomes. Both compounds released the parent drug, chlorambucil, in both experiments. The respective chemical conversion profiles of compounds **1** and **2** resulted in half-lives (*T*_1_*_/2_*) of 167 and 179 min (Figure 2A), suggesting no difference in the non-enzymatic conversion rate between the ester and amide derivatives. By contrast, the enzymatic conversion in human liver microsomes resulted in more rapid conversion of compound **1** (*T*_1_*_/2_*: 5 min) compared to compound **2** (*T*_1_*_/2_*: 52 min) (Figure 2B).

### 2.3. LAT1 Binding Affinity

**Figure 3:**
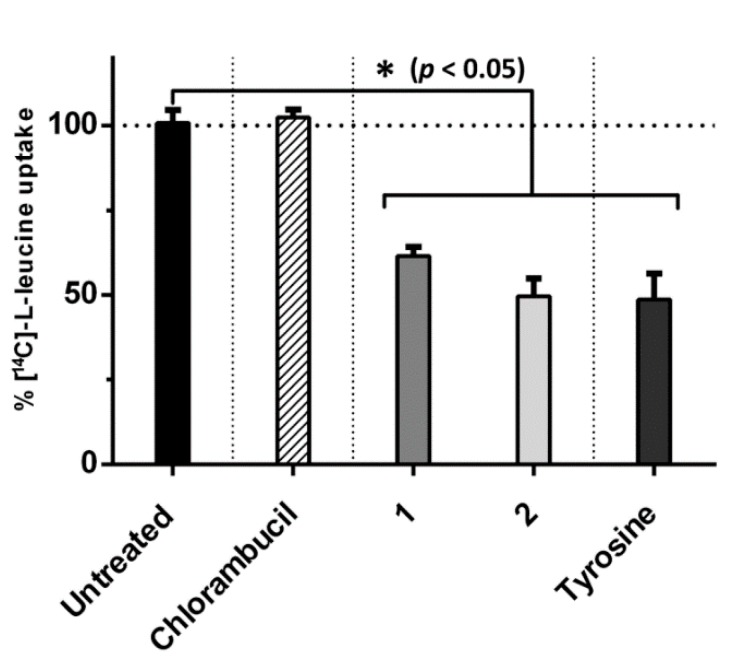
Ability of chlorambucil and its derivatives **1** and **2** (10 µM) to inhibit uptake of [^14^C]-l-leucine (0.157 µM) in MCF-7 cells. All data presented as mean ± SD (*n* = 3). * *p* < 0.05, significant difference compared to untreated control.

The binding abilities of compounds **1** and **2** to LAT1 were investigated in MCF-7 cells at 10 µM in a competitive inhibition assay with the endogenous LAT1 substrate, l-leucine. Chlorambucil, which is not a LAT1 substrate, displayed no binding affinity to LAT1 as it does not have the physicochemical facets to inhibit [^14^C]-l-leucine uptake. The chlorambucil derivatives **1** and **2**, however, exhibited binding affinity to LAT1, evidenced by the significantly decreased cellular uptake of [^14^C]-l-leucine to 61.5% ± 2.7% and 49.6% ± 5.3%, respectively. The endogenous LAT1 substrate, l-tyrosine, was able to decrease the cellular uptake of [^14^C]-l-leucine to 48.6% ± 7.9% (Figure 3).

### 2.4. In Vitro Cellular Uptake

The respective cellular uptake of compounds **1** and **2** as well as the parent drug, chlorambucil, were evaluated in MCF-7 cells to determine the exposure effect of both time and concentration on cellular uptake. Chlorambucil and its derivatives **1** and **2** were all taken up into MCF-7 cells in both a time- and concentration-dependent manner.

**Figure 4:**
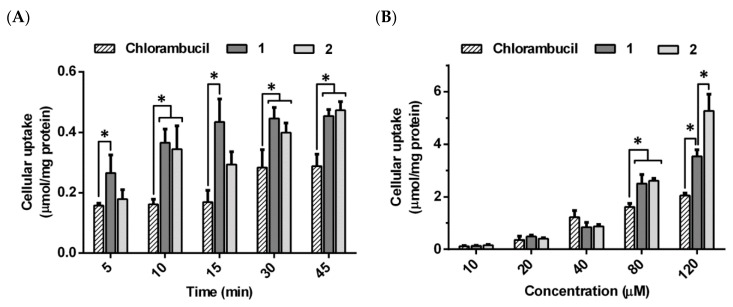
Cellular uptake of chlorambucil and its derivatives **1** and **2** at different incubation times (20 µM) (**A**) and different concentrations (15 min) (**B**) in MCF-7 cells. Values expressed as mean ± SD (*n* = 3). * *p* < 0.05, significant difference compared to chlorambucil at relevant times and concentrations.

In the time-dependent study, the uptake of compound **1** was significantly greater than that of chlorambucil at all five time points between 5 and 45 min. Compound **2** showed significantly greater cellular uptake than chlorambucil after 10 min of incubation (Figure 4A). The cellular uptake of compounds varied as follows: chlorambucil 0.16–0.29 µmol/mg of protein; compound **1** 0.27–0.45 µmol/mg of protein, and compound **2** 0.18–0.47 µmol/mg of protein.

According to time-dependent data, the uptake of both compounds **1** and **2** reached their maximum at 15 min. Therefore, 15 min was selected for the concentration-dependent uptake study as it displayed unsaturated uptake with maximum values. The respective uptakes of compounds **1** and **2** (2.5 ± 0.35 and 2.61 ± 0.10 µmol/mg of protein) were significantly higher than that of chlorambucil (1.61 ± 0.14 µmol/mg of protein) at concentrations of 80 µM and higher, at 15 min incubation time (Figure 4B).

### 2.5. Antiproliferative Activity

**Figure 5:**
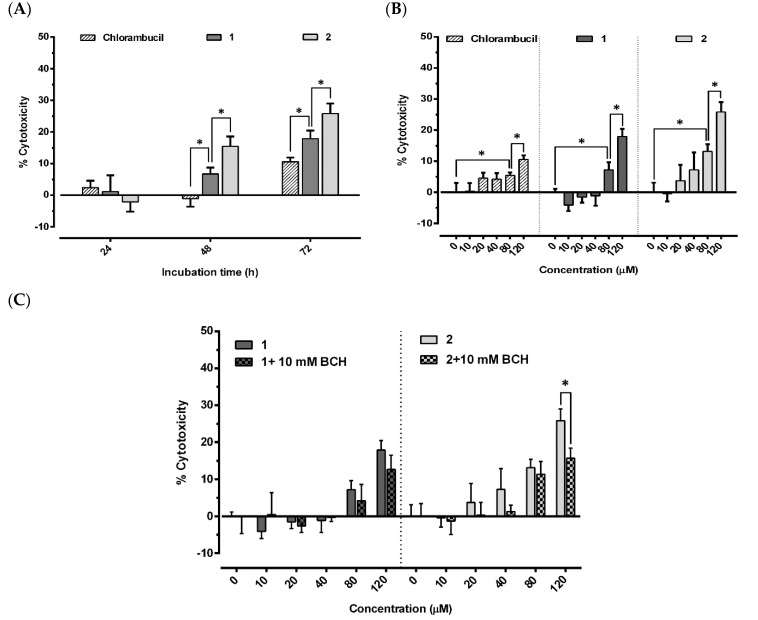
Antiproliferative activity of chlorambucil derivatives **1** and **2** at different incubation times (120 µM) (**A**), different concentrations (72 h) (**B**), and different concentrations with BCH (10 mM) (**C**) in MCF-7 cells. Results expressed as mean ± SD (*n* = 3). * *p* < 0.05, significant difference compared to chlorambucil (**A**), untreated control (**B**), and co-incubated with BCH (**C**).

The antiproliferative activity of chlorambucil and its derivatives **1** and **2** were studied in MCF-7 cells in vitro. The cell cytotoxicity was determined after treatment with all compounds at various concentrations for 24, 48, and 72 h. All compounds displayed time-dependent cell cytotoxicity (Figure 5A). There was no cell death in any of the treatment groups at 24 h incubation. At 48 h, compounds **1** and **2** displayed cell cytotoxicity in a concentration-dependent manner, while no cell death was detected in the chlorambucil-treated cells (6.7% ± 2.0%, 15.5% ± 3.1%, and (−1.1%) ± 2.5% cell cytotoxicity for **1**, **2**, and chlorambucil at 120 µM, respectively). All compounds increased cell cytotoxicity in a concentration-dependent manner at 72 h incubation with the highest cell cytotoxicity observed at 120 µM concentration. The respective cell cytotoxicity was 25.8% ± 3.2% for **2**, 18.0% ± 2.5% for **1**, and 10.6% ± 1.3% for chlorambucil (Figure 5B). In summary, compounds **1** and **2** possessed higher antiproliferative activity than chlorambucil in both a time- and concentration-dependent manner. To investigate the contribution of LAT1 on the chlorambucil derivative activity, BCH, a standard LAT1 inhibitor [17], was co-incubated with compound **1** or **2** (Figure 5C). BCH was reported to inhibit the function of LAT1 around 80%–100% with no cytotoxicity at a minimum concentration of 10 mM [18,19]. That means lower BCH concentrations (<10 mM) were not sufficient to inhibit LAT1 function, while higher concentrations (>10 mM) could be cytotoxic to the cell and interfere with the results. The reported data agreed with our study in that BCH had no significant cytotoxicity to MCF-7 (4.3% ± 3.4%) at the concentration used (10 mM, 72 h). Therefore, this concentration was used in co-incubation with compounds **1** and **2**. Our result showed that BCH was not able to reduce cytotoxicity of compound **1** significantly, possibly due to the insensitivity of MCF-7 to the chlorambucil derivative. A profound result was however demonstrated in the co-incubation of BCH with compound **2**, since BCH significantly reduced its cytotoxicity (15.7% ± 2.7%).

## 3. Discussion

Structural features for LAT1 recognition are prone to resemble moieties belonging to endogenous LAT1 substrates. The preferable features for LAT1 binding include (1) free amino and carboxylic acid groups; (2) a large and neutral side group (referring to an amino acid side chain); and, (3) an H-bond acceptor behind the amino acid side chain [20,21,22]. Nevertheless, the features are not obligatory as derivatization or replacement of the functional groups can maintain some affinity to LAT1 [1,21].

To ensure adequate binding affinity, compounds **1** and **2** were designed according to our 3D quantitative relationship (3d-QSAR) model of the LAT1 binding site so as to contain: (1) free amino and carboxylic acid groups; (2) an aromatic side chain; and, (3) an ester/amide linkage (H-bond acceptor). While both the ester **1** and amide **2** bonds can undergo bioconversion, the chlorambucil derivatives **1** and **2** were supposed to be active without bond cleavage because of the presence of the unconjugated *N,N*-bis-(2-chloroethyl)-amine moiety. The moiety acts as the active functional group to interact with DNA and exert its pharmacological activity. These compounds are, therefore, terminologically not prodrugs that possess no pharmacological activity [23], even though they can release chlorambucil and act like prodrugs.

Since the ability of the chlorambucil derivatives to bind to LAT1 depends on the amino acid promoiety, the stability of the ester or amide bond significantly affects LAT1 binding properties. In our results, there was no difference in the nonenzymatic conversion rates between the ester and amide derivatives, as the two compounds displayed a comparable half-life. Both compounds displayed chemical stability with a respective half-life being around 170 min, meriting further study; however, they underwent faster bioconversion in human liver microsomes, resulting in a dramatic reduction in half-life. The half-life of the bioconversion of the amide derivative **2** was up to 10 times higher compared with that of the ester **1**. As with other reports, the result indicates that the amide bond is less sensitive to enzymatic hydrolysis than the corresponding ester bond [2,24]; the ester derivative is more susceptible to enzymatic bioconversion, limiting its ability to reach the target site intact in vivo.

While chlorambucil demonstrated no binding affinity to LAT1 in MCF-7 cells, its derivatives **1** and **2** bound to LAT1 with an affinity comparable to the endogenous LAT1 substrate L-tyrosine. Nonetheless, the high-binding affinity is not obligatory to the efficient LAT1-mediated cellular uptake [25]. Smaller compounds seem to be taken up by LAT1 faster than the larger ones, at the expense of specificity and affinity [26]. By contrast, the larger molecules with high lipophilicity can bind more efficiently to LAT1, at the expense of transport capacity, resulting in compounds acting as inhibitors rather than substrates [27]. Consequently, the cellular uptake profiles as well as binding affinity data are crucial to determining whether the LAT-utilizing compound act as either an inhibitor or a substrate. Chlorambucil derivatives **1** and **2** were taken up into the MCF-7 cells with a higher intracellular concentration than chlorambucil itself in the time-dependent study. This result is consistent with the concentration-dependent study. We hypothesize that carrier-mediated transport is participating in improving the uptake of the designed derivatives via LAT1 into cancer cells. The computational analysis (Table 1) of the physicochemical properties indicates that chlorambucil is a small lipophilic molecule with a low polar surface area (PSA) that can effectively cross the cell membrane by passive diffusion [28,29], which has been demonstrated in the ascites sarcoma cell [30] and in situ rat intestine [31]. In contrast, derivatives **1** and **2** are larger molecules (in both weight and size) with a high PSA, making these derivatives less susceptible to passive diffusion across cell membranes. The higher cellular uptake of chlorambucil derivatives **1** and **2** is, thus, more likely attributable to other transportation processes than to passive diffusion. Taken together, the LAT1 binding study and the cellular uptake profile indicate the roles of derivatives **1** and **2** as LAT1 substrates.

Due to a more efficient cellular transport, compounds **1** and **2** accumulated in the MCF-7 cells more than did the parent drug, supporting the hypothesis that the antiproliferative activities of derivatives **1** and **2** have higher cytotoxicity than those of chlorambucil in both a time- and concentration-dependent manner. The incubation with BCH, a LAT1 inhibitor, could also decrease the chlorambucil derivative cytotoxicity, especially compound **2**, suggesting the contribution of LAT1 in antiproliferative activity. Our results demonstrate that the increased intracellular uptake attributed to LAT1 transport of derivatives **1** and **2** is positively related to their higher antiproliferative activity. Derivative **2** exhibited a higher intracellular concentration and cytotoxicity than derivative **1**. The metabolic process in the MCF-7 cells was probably engaged; since the amide derivative **2** was less sensitive to enzymatic bioconversion than the ester form **1**, the higher intracellular accumulation was detected after the treatment of **2**. The chlorambucil derivatives are pharmacologically active on their own and require no bioconversion, like a prodrug, so their intracellular concentrations relate directly to their pharmacological activity (i.e., antiproliferation). In accordance with the antiproliferative study, the MCF-7 cells were not as sensitive to chlorambucil, as cell cytotoxicities were not significantly increased until the concentrations of compounds **1** and **2** reached 80 µM. Nonetheless, our result demonstrated that the LAT1-targeting approach could increase cellular drug uptake, leading to the improvement of drug efficiency, and thereby indicating the possibility of using this approach in other LAT1-expressing cell lines.

The issues with drug stability, resistance, and selectivity limit clinical usage of chlorambucil [12]. The consequence of instability as well as drug resistance reduces chlorambucil intracellular levels, leading to inefficient treatment. Our results demonstrate that the chlorambucil derivatives **1** and **2** accumulate inside cells at a higher level and display a greater efficiency than their parent drug. In addition, the slow release of chlorambucil from its ester/amide derivatives could possibly retard metabolic and/or resistance deactivation [32].

The lack of selectivity, a major concern, hinders the clinical usage of chlorambucil due to serious adverse effects, i.e., myelosuppression and an increased risk of developing secondary neoplasm in normal tissues [33,34]. We propose conjugating chlorambucil to LAT1 substrate so as to increase drug selectivity. Whereas chlorambucil enters the cells nonspecifically by passive diffusion, its amino acid conjugates bypass the cell membrane using more specific active transport. Since LAT1 is upregulated in many cancers, specific transport using LAT1 as a carrier is conceivable to accumulate the chlorambucil derivatives in cancer cells at a higher level than in normal cells. Therefore, the higher selectivity could possibly lead to the reduction of nonselective side effects. In addition, the binding affinity to LAT1 could also be enhanced by structural modifications. The *meta*-conjugation of l-phenylalanine (or l-tyrosine) to a parent drug has been shown to display higher binding affinity to LAT1 than *para*-conjugation [2,26]. In our experiment, chlorambucil conjugation to l-tyrosine derivatives at the *meta*-position was highly acid sensitive, and both the ester and amide bonds were cleaved during the deprotection process (unpublished data).

LAT1 has been reported to share its substrates with other transporters such as LAT2 [35], monocarboxylate transporter 8 and 10 (MCT8/10) [36], and organic anions transporting polypeptides (OATPs) [21]. Whereas the OATPs transport a broad range of amphipathic substrates and are not specific to only amino acids, the MCT8/10 specifically uptakes thyroid hormone derivatives and displays low affinity to aromatic amino acids such as tyrosine and tryptophan (K_m_ ~ 5 mM in *Xenopus* oocyte) [37]. On the other hand, LAT1 preferentially transports large and neutral amino acids, while LAT2 displays a broader range of substrate selectivity including smaller amino acids. In general, LAT1 binds to its substrates with higher affinity (K_m_ ~ 10‒20 µM in rat) than LAT2 to its substrates (K_m_ ~ 30‒300 µM in rat) [38]. Therefore, it is reasonable to target LAT1 for improved cancer drug uptake using aromatic amino acids, such as tyrosine, as the promoiety. Based on the results, LAT1 contributed to cellular uptake of chlorambucil derivatives. However, the contribution of other amino acid transporters (OATPs, MCTs, and LAT2) should not be ruled out as they share overlapping substrates. While some of the other transporters can be found in normal tissues, the unspecific binding among these transporters could complicate LAT1 utilization of compounds by impairing their selectivity to cancer cells. The off-target effects are also possible and should be taken into consideration in further in vivo studies.

In short, our study demonstrated a drug-design approach to increase the efficiency of chlorambucil, as its derivatives displayed both higher intracellular accumulation and cytotoxicity against cancer, which compensate for the issues of drug instability and resistance that reduce intracellular concentration and efficiency. As LAT1 is overexpressed in several cancers, the structural modification of the drug could possibly increase drug selectivity and reduce nonselective side effects. We suggest that the (pro)drug design approach might be applied to other chemotherapeutic agents that contain available functional group(s) to conjugate to the LAT1 substrate, rectifying permeability and selectivity issues. Further studies are also needed to elucidate transporter selectivity and structural modifications for enhancing the selectivity of chlorambucil derivatives. The pharmacokinetic profile using an animal model should also be addressed since metabolism, unbound/bound factions, and distribution [39] could determine the efficiency of the derivatives in vivo.

## 4. Materials and Methods

### 4.1. General Synthetic Procedure

The amino acid component of compounds **1** and **2** were synthesized from Boc-l-tyrosine-O*t*Bu (Combi-Blocks, San Diego, CA, USA) and Boc-4-nitro-l-phenylalanine (Combi-Blocks), respectively. Chlorambucil was obtained from TCI (Tokyo, Japan). *N*-Ethyl-*N*′-(3-dimethylaminopropyl)carbodiimide (EDC), 4-(dimethylamino)pyridine (DMAP), and Pd (10% on activated charcoal), potassium carbonate (K_2_CO_3_), dimethyl sulfate ((CH_3_O)_2_SO_2_), and celite were purchased from Sigma-Aldrich (St. Louis, MO, USA).

Thin-layer chromatography (TLC) with aluminum sheets coated with silica gel 60 F_245_ (0.24 mm) (Merck, Darmstadt, Germany) was used with suitable visualization to monitor reactions. Flash chromatography with the Sepacore Flash system, coupled with Sepacore flash cartridge (Buchi AG, Flawil, Switzerland), was used for purification. Nuclear magnetic resonance (NMR) spectra were recorded by Bruker Avance 500 spectrometer (Bruker Biospin, Fällanden, Switzerland), operating at 500.13 MHz for ^1^H and at 125.75 MHz for ^13^C using tetramethylsilane (TMS) as an internal standard. Mass spectra were characterized using a Finnigan LCQ quadrupole ion trap mass spectrometer (Finnigan MAT, San Jose, CA, USA) coupled with an electrospray ionization source. Purity of final products was confirmed by quantitative ^1^H NMR (qHNMR) using maleic acid as an internal standard.

**Boc-4-amino-l-phenylalanine methyl ester 6:** Boc-4-nitro-l-phenylalanine **4** (400 mg, 1.28 mmol) was dissolved in 10 mL acetone after which K_2_CO_3_ (884 mg, 6.4 mmol) and (CH_3_O)_2_SO_2_ (242 µL, 2.56 mmol) were added. The reaction was stirred at room temperature overnight. The reaction mixture was subsequently evaporated under reduced pressure to get a dry residue of Boc-4-nitro-l-phenylalanine methyl ester (378 mg). ^1^H NMR (MeOD): δ ppm 1.38 (s, 9H), 3.03–3.07 (m, 1H), 3.27–3.31 (m, 1H), 3.74 (s, 3H), 4.46 (dd, *J*_1_ = 5.3 Hz, *J*_2_ = 9.2 Hz), 7.49 (d, *J* = 8.4 Hz, 2H), 8.18 (d, *J* = 8.4 Hz, 2H). Pd (10% on activated charcoal) (10 mg) was carefully added to the solution of Boc-4-nitro-l-phenylalanine methyl ester (378 mg, 0.58 mmol) in methanol (30 mL). The reaction was stirred in a H_2_-saturated atmosphere (344.7 kPa (50 psi)) overnight. The mixture was then filtered through celite and evaporated under reduced pressure. The residue was purified by flash chromatography in gradient mode (petroleum ether/ethyl acetate=1:99 to 99:1) to get compound **6** (165 mg, 43%), a brown solid. ^1^H NMR (MeOD): δ ppm 1.41 (s, 9H), 2.79–2.83 (m, 1H), 2.94–2.98 (m, 1H), 3.69 (s, 3H), 4.27–4.30 (m, 1H), 6.67–6.69 (d, *J* = 8.2 Hz, 2H), 6.94–6.96 (d, *J* = 8.2 Hz. 2H).

**Chlorambucil (Boc-O*t*Bu-phenylalaninate) ester 7:** Chlorambucil **3** (200 mg, 0.66 mmol) was dissolved in 5 mL dichloromethane (DCM) with DMAP (154 mg, 0.8 mmol) and Boc-l-tyrosine-O*t*Bu **5** (270 mg, 0.8 mmol). EDC (98 mg, 0.8 mmol) in 5 mL DCM was then added dropwise into the mixture and the reaction stirred at room temperature overnight. Ethyl acetate (40 mL) was subsequently added to the reaction mixture. The combined solvent was washed with 1 M hydrochloric acid (HCl) (10 mL × 2), saturated sodium bicarbonate (NaHCO_3_) (10 mL × 2), and H_2_O (10 mL), respectively. The organic solvent was dried over anhydrous sodium sulfate (Na_2_SO_4_), filtrated, and evaporated under reduced pressure. The reaction yielded compound **7** (270 mg, 88%), an off-white solid. ^1^H NMR (CDCl_3_): δ ppm 1.40 (s, 9H), 1.43 (s, 9H), 2.03 (quin, *J* = 7.5 Hz, 2H), 2.55 (t, *J* = 7.5 Hz, 2H), 2.64 (t, *J* = 7.5 Hz, 2H), 3.04 (d, *J* = 5.7 Hz, 2H), 3.61-3.72 (m, 8H), 4.43 (m, 1H), 5.00 (d, *J* = 8.5 Hz, 1H), 6.65 (d, *J* = 8.4 Hz, 2H), 6.99 (d, *J* = 8.4 Hz, 2H), 7.11 (d, *J* = 8.4 Hz, 2H), 7.17 (d, *J* = 8.4 Hz, 2H). ^13^C NMR (CDCl_3_): δ ppm 26.73 (1×C), 27.96 (3×C), 28.33 (3×C), 33.67 (1×C), 33.94 (1×C), 40.49 (2×C), 53.66 (2×C), 54.80 (1×C), 79.75 (1×C), 82.20 (1×C), 112,29 (2×C), 121.39 (2×C), 129.77 (2×C), 130.47 (2×C), 134.07 (1×C), 144.37 (1×C), 149.54 (1×C), 170.80 (1×C), 172.01 (1×C).

**Chlorambucil (Boc-methyl-phenylalaninate) amide 8:** Chlorambucil **3** (100 mg, 0.33 mmol) was dissolved in 5 mL DCM with DMAP (77 mg, 0.4 mmol) and compound **6** (130 mg, 0.4 mmol). EDC (49 mg, 0.4 mmol) in 5 mL DCM was then added dropwise into the mixture and the reaction stirred at room temperature overnight. Ethyl acetate (40 mL) was then added into the reaction mixture. The combined solvent was washed with 1 M HCl (10 mL × 2), saturated NaHCO_3_ (10 mL × 2), and H_2_O (10 mL), respectively. The organic solvent was dried over anhydrous Na_2_SO_4_, filtrated, and evaporated under reduced pressure. The reaction yielded compound **8** (118 mg, 77%), a brown solid. ^1^H NMR (CDCl_3_): δ ppm 1.42 (s, 9H), 2.02 (quin, *J* = 7.3 Hz, 2H), 2.33 (t, *J* = 7.5 Hz, 2H), 2.62 (t, *J* = 7.3 Hz, 2H), 3.00–3.11 (m, 2H), 3.61–3.72 (m, 11H), 4.55 (m, 1H), 4.95 (d, *J* = 7.7 Hz, 1H), 6.63 (d, *J* = 8.5 Hz, 2H), 7.04–7.10 (m, 4H), 7.42 (d, *J* = 8.5 Hz, 2H). ^13^C NMR (MeOD): δ ppm 19.44 (1×C), 27.25 (3×C), 33.34 (1×C), 35.91 (1×C), 40.29 (2×C), 51.19 (1×C), 53.17 (2×C), 60.12 (1×C), 79.23 (1×C), 112,16 (2×C), 119.83 (2×C), 129.17 (2×C), 129.26 (2×C), 130.31 (1×C), 132.62 (1×C), 144.62 (1×C), 172.78 (1×C), 173.00 (1×C).

**Chlorambucil phenylalanine ester 1:** Compound **7** (255 mg, 0.55 mmol) was dissolved in DCM (4 mL) and trifluoroacetic acid (2 mL) was added dropwise. The reaction was stirred for 6 h at room temperature and the solvent evaporated. The residue was purified by flash chromatography in gradient mode (DCM/MeOH=1:99 to 99:1) to get compound **1**, an off-white solid in trifluoroacetic salt (2X) form (134 mg, 35%). ^1^H NMR (MeOD): δ ppm 2.00 (quin, *J* = 7.2 Hz, 2H), 2.58 (t, *J* = 7.3 Hz, 2H), 2.64 (t, *J* = 7.4 Hz, 2H), 3.10–3.14 (dd, *J*_1_ = 8.2 Hz, *J*_2_ = 14.8 Hz, 1H), 3.32–3.34 (m, 1H), 3.65–3.75 (m, 8H), 4.08 (dd, *J*_1_ = 8.2 Hz, *J*_2_ = 5.1 Hz, 1H), 6.71 (d, *J* = 8.7 Hz, 2H), 7.06 (d, *J* = 8.5 Hz, 2H), 7.11 (d, *J* = 8.6 Hz, 2H), 7.17 (d, *J* = 8.5 Hz, 2H). ^13^C NMR (MeOD): δ ppm 26.43, 32.88, 33.55, 35.58, 40.30 (2×C), 53.17 (2×C), 54.69, 112.17 (2×C), 122.01 (2×C), 129.30 (2×C), 129.99, 130.22 (2×C), 132.61, 144.75, 150.34, 170.72, 172.80. MS: calcd. for C_23_H_28_Cl_2_N_2_O_4_ 466.14 [M]^+^, found 467.12 [M+H]^+^, 97% purity.

**Chlorambucil phenylalanine amide 2:** Compound **8** (100 mg, 0.21 mmol) was dissolved in tetrahydrofuran (5 mL) and lithium hydroxide (20 mg, 0.8 mmol) in H_2_O (5 mL) added dropwise. The reaction was stirred for 1.5 h and the solvent evaporated. The intermediate compound was then dissolved in DCM (4 mL) and trifluoroacetic acid (2 mL) was added dropwise. The reaction was stirred for 2 h at room temperature and the solvent evaporated. The residue was purified by flash chromatography in gradient mode (DCM/MeOH=1:99 to 99:1) and recrystallized using MeOH to get compound **2**, a light-brown solid in trifluoroacetic salt (2X) form (24 mg, 16%). ^1^H NMR (MeOD): δ ppm 1.98 (quin, *J* = 7.4 Hz, 2H), 2.38 (t, *J* = 7.5 Hz, 2H), 2.61 (t, *J* = 7.4 Hz, 2H), 2.98–3.02 (dd, *J*_1_ = 14.4 Hz, *J*_2_ = 8.8 Hz, 1H), 3.28–3.33 (dd, *J*_1_ = 4.1 Hz, *J*_2_ = 14.9 Hz, 1H), 3.65–3.75 (m, 8H), 3.77–3.79 (dd, *J*_1_ = 4.1 Hz, *J*_2_ = 8.4 Hz, 1H), 6.70 (d, *J* = 8.6 Hz, 2H), 7.10 (d, *J* = 8.6 Hz, 2H), 7.27 (d, *J* = 8.4 Hz, 2H), 7.52 (d, *J* = 8.4 Hz, 2H). ^13^C NMR (MeOD): δ ppm 27.34, 33.82, 35.86, 36.24, 40.28 (2×C), 53.17 (2×C), 56.10, 112.17 (2×C), 120.48 (2×C), 129.24 (2×C), 129.39 (2×C), 130.28, 131.43, 137.72, 144.65, 172.28, 173.19. MS: calcd. for C_23_H_29_Cl_2_N_3_O_3_ 465.16 [M]^+^, found 466.09 [M+H]^+^, 95% purity.

### 4.2. HPLC Analysis

The analytical and biological samples were injected into a reversed-phase C18 analytical column Zorbax SB-C18 (3 × 150 mm, 3.5 µm) coupled with guard-column Zorbax SB-C18 (Agilent Technologies, Little Falls Wilmington, DE, USA). The solutions were eluted in isocratic mode with 50% acetonitrile and deionized water (with 0.1% formic acid) as eluents at a flow rate of 0.8 mL/min. The sample peaks were monitored by Agilent 1100 series HPLC (Agilent Technologies, Waldbronn, Karlsruhe, Germany) with UV detection at 254 nm. The retention time of chlorambucil derivatives (**1** and **2**) and chlorambucil (**3**) were 2.4, 1.6, and 5.0 min, respectively.

### 4.3. In Vitro Conversion

The rates of chemical conversion of compounds **1** and **2** were studied in aqueous phosphate buffer solutions (50 mM, pH 7.4) at 37 °C. The solutions of the chlorambucil derivatives were prepared by adding 80 µL of stock solution (0.5 mg/mL in 5% HP-β-cyclodextrin (Carvasol, Wacker Chemie, Munich, German) in phosphate buffer) to 720 µL of the preheated buffer. At predetermined time intervals, aliquots (50 μL) were sampled, mixed with MeOH (50 µL), and analyzed for the remaining compound by HPLC.

Compounds **1** and **2** were incubated with the human liver microsomes as an indication of the susceptibility to bioconversion in the liver. The reactions were initiated by adding 3.0 µL of compounds (10 mM in DMSO) to a preheated solution consisting of 747 µL phosphate buffer (20 mM, pH 7.4), 200 µL NADPH (1 mM), and 50 µL human liver microsomes (20 mg/mL). The mixture was incubated thermostatically at 37 °C, and the 50 µL aliquots were withdrawn at predetermined time intervals. The aliquots were then immediately mixed with 100 µL of ice cold MeOH and centrifuged at 14,000× *g* for 5 min to precipitate proteins. The clear supernatants were analyzed for the remaining compound by HPLC. The respective pseudo-first-order half-lives (*T*_1__/2_) for the conversion rate of the compounds were calculated from the slope of the linear portion of the remaining compound logarithm plots against time.

### 4.4. Cell Line and Culture Condition

The human breast adenocarcinoma cell line MCF-7 was purchased from the American Type Culture Collection (ATCC#HTB-22) (Manassas, VA, USA). The MCF-7 cells were cultured in Dulbecco’s Modified Eagle Medium (DMEM) supplemented with 2 mM l-glutamine, 10% fetal bovine serum (FBS), 50 units/mL penicillin, and 50% streptomycin. The cell line was incubated in a humidified incubator at 37 °C with 5% CO_2_, and the cells were used after reaching approximately 80% confluency.

### 4.5. LAT1 Binding Affinity

The expression and function of LAT1 and LAT2 in MCF-7 cells was characterized in our recent study [26]. The study indicated that LAT1 is predominant in both expression and function compared to LAT2, implying its role as a major transporter responsible for the uptake of LAT1 substrates. The ability of compounds to bind to LAT1 was determined as previously described [40]. In brief, MCF-7 cells were seeded into collagen-coated, 24-well plates at a density of 1.5 × 10^5^ cells/well. The seeded cells were incubated overnight in the incubator before the affinity experiment. The cells were then pre-incubated in 500 µL of warm HBSS (Hank’s balance salt solution) for 10 min. After the removal of HBSS, the cells were incubated for 5 min in 250 µL of 5% HP-β-cyclodextrin in HBSS, containing 0.157 µM of [^14^C]-l-leucine (PerkinElmer, Waltham, MA, USA) and 10 µM of compound: chlorambucil, its derivatives (**1** and **2**), or l-tyrosine (Fluka, Buchs, Switzerland). The cells were washed twice with ice-cold HBSS (500 µL) on an ice bath and lysed by 250 µL of 0.1 M sodium hydroxide (NaOH) for 1 h at room temperature. The lysate was then mixed with 1 mL of an emulsifier-safe cocktail (PerkinElmer) and the radioactivity recorded by liquid scintillation counter (Wallac 1450 MicroBeta, Wallac Oy, Finland).

### 4.6. In Vitro Cellular Uptake

Determination of the intracellular concentration of compounds was performed following a previous report [25]. Briefly, MCF-7 cells were seeded and treated as in LAT1 affinity studies. The uptake of chlorambucil and its derivatives was studied in 250 µL of 5% HP-β-cyclodextrin in HBSS by incubating the MCF-7 cells for varying incubation times (5–45 min, 20 µM) or concentrations (10–120 µM, 15 min). The cells were washed twice with ice-cold HBSS (500 µL) in an ice bath, then lysed by 1% perchloric acid (250 µL) for 0.5 h at room temperature. The suspension was removed to an Eppendorf tube and centrifuged at 14,000× *g*, 4 °C, for 5 min. The supernatant (200 µL) was analyzed by HPLC. The respective standard curve for the intracellular concentration of compounds (**1**, **2**, or **3**) was performed by spiking known amounts of the compounds into cell lysate of untreated cells. For protein determination, the cells were lysed using 250 µL of 0.1 M NaOH for 0.5 h instead of 1% perchloric acid and were determined using Bio-Rad protein assay (Bio-Rad, CA, USA) as described by the manufacturer, using serum albumin (Sigma-Aldrich) as the standard. The protein concentrations in samples were recorded using an EnVision multimode plate reader (PerkinElmer). The final results were expressed as the intracellular concentration normalized by its protein content.

### 4.7. Antiproliferative Activity

Cell cytotoxicity was assessed using MTT (3-(4,5-Dimethylthiazol-2-yl)-2,5-diphenyltetrazolium bromide) assay as previously described [41]. MCF-7 cells were seeded at a density of 6 × 10^3^ cells/well into a 96-well plate and incubated overnight in an incubator. Thereafter, chlorambucil and its derivatives were added and incubated for 24, 48, and 72 h with a final concentration ranging from 10 to 120 µM. At predetermined times, MTT (Amresco, Solon, OH, USA) solution, in phosphate buffer saline, was added to each well to achieve a final concentration of 0.5 mg/mL. The cells were then incubated for 3 h in an incubator before discarding the media, after which 100 µL of DMSO were added to each well to solubilize the formazan. The absorbance of solubilized formazan was measured at 570 nm (reference wavelength: 650 nm) using an EnSight multimode plate reader (PerkinElmer). BCH (2-aminobicyclo-[2,2,1]-heptane-2-carboxylic acid) (Sigma-Aldrich) was used as a standard LAT1 inhibitor in the co-incubation assay. DMSO in 5% HP-β-cyclodextrin was used as the dispersing agent, which had no cytotoxicity at the respective concentrations at any of the predetermined time points.

### 4.8. Computational Analysis

The chemical structures were analyzed using CS ChemBio3D Ultra (version 12.0; Cambridge Soft Corporation, Waltham, MA, USA). For each molecule, energy minimization was performed by molecular mechanics (MM2) with a root-mean-square of 0.001. The property server was used to compute the log P, molecular weight, molecular accessible area (Å^2^) (as per the Connolly method), and polar surface area (PSA) (Å^2^).

### 4.9. Statistical Analysis

The data were analyzed for normality of distribution (Shapiro–Wilk test) and homogeneity of variance (Levene’s test) (*p* > 0.05). Differences among treatments were assessed using a one-way ANOVA followed by a Tukey’s multiple comparison post hoc tests using SPSS 19.0 software for Windows^®^ (SPSS Inc, Chicago, IL, USA). Respective data were presented as a mean ± SD and any differences with a *p* value < 0.05 were considered statistically significant.

## 5. Conclusions

Structural modifications of a chemotherapeutic drug by conjugating it to LAT1 substrate can increase its effectiveness against cancer. A synthetic method for conjugating chlorambucil and tyrosine via either an ester or amide bond was developed to obtain derivatives **1** and **2**. The MCF-7 cell line was used as a cancer cell model to determine binding ability, intracellular uptake, and antiproliferative activity in vitro. The chlorambucil derivatives bound to LAT1 in a manner comparable to l-tyrosine. The respective intracellular concentration after treatment with derivatives **1** and **2** was higher and their respective antiproliferative activity had a higher cytotoxicity than that of chlorambucil. By exploiting LAT1 as a carrier-mediated transporter, derivatives **1** and **2** efficiently permeated the cell membrane, accumulated, and exerted their cytotoxicity in vitro more efficiently than did chlorambucil. Our results support rational drug design to elevate the intracellular drug level using a specific transporter as well as enhancing its chemotherapeutic efficiency.

## Figures and Tables

**Table 1:** Physicochemical properties of chlorambucil and its derivatives **1** and **2.**

Computational Parameter	Compound		
	Chlorambucil	1	2
Log P	4.1	4.7	4.1
Molecular weight (g/mol)	304.2	467.4	466.4
Molecular accessible area (Å^2^)	558.3	798.7	802.6
Polar surface area (Å^2^)	40.5	92.8	95.7
